# Dr. B. R. Johnson

**Published:** 1899-02

**Authors:** 


Dr. B. R. Johnson, of Gowanda, N. Y., died in that village January 21, 1899. Dr. Johnson had been ill some weeks suffering from pneumonia and his death had been expected for several days. Yet, when the end came the shock was a severe one to the community.

Dr. Johnson was held in the highest esteem by the people of Gowanda and vicinity. He was a popular man, an active practitioner, devoted to his profession and ready to sacrifice his personal comfort at all times to heed the calls of the sick. Dr. Johnson's father, also a physician, died at the family home at Gowanda last summer. The deceased leaves a widow and two children. He was a brother of Dr. Burt C. Johnson, of Buffalo.

